# Randomized controlled trial of asynchronous vs. synchronous online teaching formats: equal knowledge after training, greater acceptance and lower intrinsic motivation through asynchronous online learning

**DOI:** 10.1186/s12909-025-07481-4

**Published:** 2025-06-19

**Authors:** Monika Zsifkovits, Lukas Amplatz, Nina Triebner, Janine Utz, Johannes Kornhuber, Philipp Spitzer

**Affiliations:** 1 Department of Psychiatry and Psychotherapy, Friedrich-Alexander-Universität Erlangen-Nürnberg, Schwabachanlage 6, 91054 Erlangen, Germany; 2 Department of Styria, Austrian Red Cross, Merangasse 26, Graz, 8010 Austria

**Keywords:** Online learning, Asynchronous online learning, Synchronous online learning, Intrinsic motivation, Self-determination theory

## Abstract

**Background:**

The growing importance of online education in recent years has led to an increased focus on implementing and optimizing online learning formats. This study investigated how a lecture delivered in an asynchronous or synchronous online teaching format affects acceptance, intrinsic motivation and knowledge levels after training. The results can be used to optimize online education by identifying format-specific advantages and adapting them to learners’ needs.

**Methods:**

All the Styrian paramedics (*N* = 5910) were invited to participate in the study and randomly assigned to one of two groups. A total of 1044 participants completed the trial, with one group receiving asynchronous training via a learning platform (*N* = 545) and the other group participating in synchronous training via webinars (*N* = 499) providing the same content. After completing a two-hour psychiatric emergency course, the participants were invited to complete a multiple-choice test and a survey assessing acceptance, preferences and intrinsic motivation. Linear regression, t tests and mediation analyses were conducted.

**Results:**

The asynchronous training format was significantly more accepted (*p* <.001) and preferred overall. The participants’ preferences depended significantly on the type of learning format (*p* <.001). The synchronous learning format fostered greater intrinsic motivation (*p* =.001) and greater perceived autonomy (*p* <.001) but also a higher level of perceived pressure/tension (*p* =.003). The analysis revealed no significant difference in test results (*p* =.449) or perceived competence between the groups (*p* =.420). Furthermore, the difference in intrinsic motivation was fully mediated by perceived autonomy.

**Conclusions:**

There are different advantages and disadvantages to providing a lecture via webinar or online learning platform. Both formats are equally effective in terms of knowledge levels after training, highlighting the importance of adapting teaching strategies to learners’ preferences.

**Supplementary Information:**

The online version contains supplementary material available at 10.1186/s12909-025-07481-4.

## Background

The importance of online teaching in pre- and postgraduate education has increased considerably in recent years [1]. In particular, interest in the use of online teaching methods among educational institutions has been pronounced in the postpandemic era, given the numerous benefits that such methods offer [2]. These advantages include increased student motivation, enhanced memory retention, and access to diverse multimedia content [3]. Online education has also reduced operational costs and promoted learner-centered approaches through flexible study options and active participation [4]. Studies in the medical field have shown that online teaching is as effective as traditional methods in terms of knowledge and skills acquisition while resulting in greater student satisfaction [5, 6]. In many practical settings, such as continuing professional development, institutions are now faced with the challenge of choosing between different online teaching formats. This decision has implications for scheduling, resource planning and learner engagement.

Online teaching can be organized in a variety of ways, with a distinction between synchronous and asynchronous teaching formats. Asynchronous learning formats allow students to access learning materials and complete tasks on their own schedule (e.g., on a learning platform). In contrast, synchronous learning formats involve real-time interaction between educators and students through live sessions or discussions (for example, in webinars) [7]. Importantly, both synchronous and asynchronous online teaching formats can be implemented in highly diverse ways, depending on the underlying instructional design. For example, synchronous formats such as webinars may include interactive case-based discussions, real-time feedback mechanisms, or collaborative group work [8]. Asynchronous settings could incorporate gamification elements with the intention of enhancing engagement [9]. In our study, we focus on two common applications: recorded lectures on a learning platform, which learners can review at any time and where participants work individually on case studies, and live webinars, which enable real-time interaction between instructors and participants and include breakout rooms for group discussions on the same case studies. Both modes have advantages and disadvantages: synchronous learning promotes immediate feedback and collaboration, whereas asynchronous learning offers flexibility and can accommodate diverse schedules [10]. It is therefore necessary to examine the differences between the two online teaching formats in greater detail to implement the most suitable learning format and to better understand the different advantages and disadvantages.

Although online learning offers several advantages, it is not a universally accepted method of education. For example, as demonstrated in the study conducted by Riley et al., emergency physicians may reject online learning [11]. Previous research has suggested that synchronous online learning is generally more accepted and preferred by students, as it provides real-time interaction and is perceived to increase engagement and learning effectiveness compared with asynchronous formats [12]. Furthermore, research indicates that acceptance of the online teaching format is significantly associated with enhanced learning outcomes [13]. Consequently, it is equally important to examine the acceptance of different online learning formats among learners and to employ the preferred online learning format in appropriate settings.

In (online) education, learning success is an important measure of effectiveness. A meta-analysis by Martin et al. (2021) revealed small differences in learning outcomes between synchronous and asynchronous online formats, with synchronous learning showing a slight advantage in knowledge acquisition [14]. Furthermore, research indicates that factors such as pressure, intrinsic motivation and perceived competence significantly influence learning outcomes. For example, elevated pressure has been demonstrated to increase anxiety, thereby negatively impacting learning performance [15]. Conversely, heightened perceived competence has been shown to enhance self-regulated learning and intrinsic motivation, leading to improved learning outcomes [16]. A more detailed examination of different online learning environments is essential to assess their effectiveness accurately. Investigating the impact of various factors, such as perceived pressure and competence, on test results will provide valuable insights into optimizing online instructional formats and increasing student success.

Intrinsic motivation, defined as the internal drive to pursue activities for personal satisfaction, is essential for effective learning [17, 18]. Learners who are intrinsically motivated have been shown to engage more deeply, demonstrate resilience in the face of challenges, and achieve better outcomes [17–19]. This is especially important in online learning, where the lack of direct supervision requires learners to self-regulate their engagement. Self-determination theory (SDT) posits that intrinsic motivation is contingent on the satisfaction of an individual’s basic psychological needs for autonomy, perceived competence, and relatedness [17]. According to Ryan and Deci, intrinsic motivation is associated not only with enhanced cognitive flexibility, a more positive emotional state and heightened creativity but also with superior learning outcomes and greater acceptance of the learning format [17]. A systematic review revealed that fostering these needs, along with self-regulated learning and meaningful feedback, significantly enhances intrinsic motivation and learning success [20]. Recent research highlights the value of SDT in online higher education and recommends further exploration of its role in digital learning [21]. Synchronous teaching formats have been shown to enhance intrinsic motivation by providing immediate interaction and real-time feedback, fulfilling learners’ needs for competence and relatedness [21]. Fabriz et al. (2021) reported that students in synchronous courses reported significantly greater support for these needs and greater satisfaction than did those in asynchronous settings [21]. To achieve a better understanding of these dynamics, it is essential to explore the application of SDT in online education to better understand the interactions between its key components and their impact on learning experiences and knowledge levels after training.

This study addresses a research gap concerning the factors that influence acceptance, intrinsic motivation and knowledge status after training, which are of particular importance in the context of the growing importance of online teaching formats in the future. The selection of these variables was because they capture both affective (motivation and acceptance) and cognitive (knowledge status) outcomes that are central to understanding online learning effectiveness. These constructs directly reflect the predictions derived from SDT and have been widely reported in the literature as critical determinants of successful learning.

The study was conducted among employees of the Styrian ambulance service, as paramedics are a highly diverse group (in terms of education, age, experience, profession, etc.), and the results are therefore applicable to a broad population. The effectiveness of online learning is significantly influenced by the quality of instruction. Pedagogical styles and designs can vary considerably in both synchronous and asynchronous learning environments. For example, an asynchronous environment that incorporates gamified elements or interactive multimedia can foster high intrinsic motivation and engagement [22]. A key practical question is whether it is more effective to deliver lecture content as a live webinar, which enables real-time interaction, or as recorded lectures on a learning platform, which offers maximum flexibility and self-paced review. To address this question, the present study was designed to create learning environments that are as similar as possible in both synchronous and asynchronous formats, providing learners with comparable content and opportunities for engagement to enable more meaningful comparisons.

**Hypotheses**:

1. **Acceptance and preferences**.
  1.1. The acceptance of live webinars is superior to that of recorded lectures on a learning platform.
  1.2. The live webinar is preferred over recorded lectures on a learning platform.
  1.3. Intrinsic motivation significantly influences the acceptance of the learning format.
2. **Knowledge level**.
  2.1. Live webinars lead to higher test scores than recorded online lectures do.
  2.2. Higher perceived pressure/tension decreases test results, whereas higher perceived competence, intrinsic motivation and perceived choice lead to better test results in online learning environments.
3. **Intrinsic motivation**.
  3.1. Intrinsic motivation is greater among participants in live webinars than among those watching recorded online lectures.
  3.2. The perceived freedom of choice and perceived competence of live webinars exceed those of participants watching recorded online lectures on the learning platform.
  3.3. Higher perceived freedom of choice and perceived competence lead to greater intrinsic motivation, whereas higher pressure and tension lead to lower intrinsic motivation.

## Methods

### Participants, ethics and study design

**Fig. 1:**
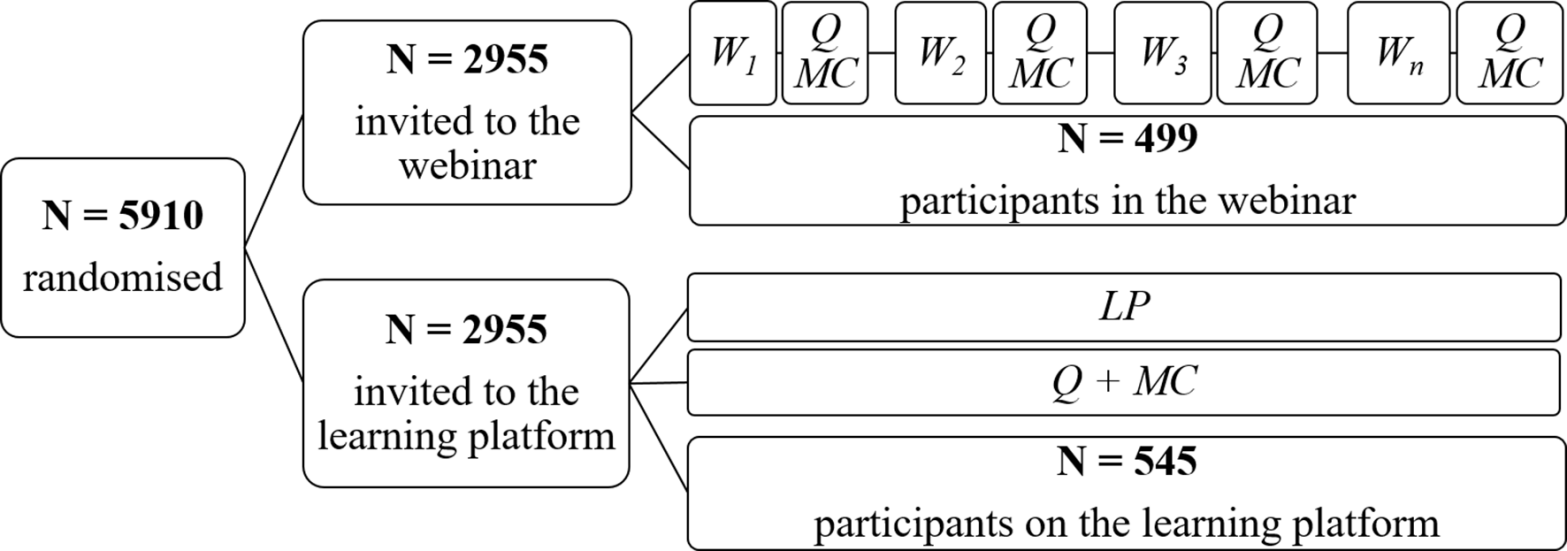
Study design: All paramedics (*N* = 5910) were invited to participate in the study and were randomly assigned to one of the two study groups. The participants were required to attend a two-hour training course either through one of the seven webinars (W_1--_W_7_) or through the learning platform (LP). The participants were subsequently invited to complete an anonymous online survey after the training course. A total of 1044 people completed the study, with 499 participating in the webinar and 545 completing the training on the LP. In addition to demographic data, the questionnaire (Q) included questions examining intrinsic motivation and acceptance and 20 multiple-choice (MC) questions related to the training content

The Austrian Emergency Medical Service (EMS) utilizes a mixed system, employing both full-time and volunteer paramedics. In accordance with the legislation, all Austrian paramedics are obliged to complete 16 h of further training every two years. To meet this requirement, the Austrian Red Cross provides four further training sessions per year, each lasting two hours. All the Styrian paramedics were randomly assigned to either watch prerecorded lectures (via the “Moodle” online learning platform of the Austrian Red Cross) or attend a live webinar (via Microsoft^®^ Teams), presenting the same content. Randomization was performed via the “RANDBETWEEN function” in Microsoft^®^ Excel. Afterward, the paramedics were asked to participate in mandatory training between January and February 2024. At the end of the training, the participants were invited to complete an anonymous survey and a multiple-choice test on a voluntary basis (Fig. 1). The study was conducted in accordance with the Declaration of Helsinki. All participants provided their informed consent to participate in the research, did so voluntarily, and were explicitly informed that their participation would not impact the recognition of the course and that the data they provided would be processed anonymously. The study was approved by the Ethics Committee of Friedrich-Alexander University Erlangen-Nuremberg (Ref. Nr.: 23-315-B).

### Course concept

The training session lasted two hours and included a “psychiatric emergency” as the focal subject. The training encompassed a range of topics relevant to the field of psychiatry, including case studies, psychiatric disorders, social aspects, communication skills and judicial aspects.

For the webinar, seven training dates were made available between January and February 2024. The total number of webinar participants ranged from 40 to 120. The lecture combined theoretical input from a lecturer and intermittent group discussions. The webinar participants engaged in breakout room discussions of case studies in small groups of four to six people, after which they presented their proposed solutions to the other participants.

The learning platform, which included video recordings of the lecture, was accessible on a 24/7 basis throughout January and February 2024. The lecture comprised a combination of theoretical input from the lecturer followed by case studies to facilitate the participants’ learning process. The participants using the learning platform worked on the same case studies as the webinar group but were required to work independently. They subsequently answered multiple-choice questions about the case studies.

The training content was largely identical for both groups. To facilitate a more meaningful comparison between the two groups, the presentations were delivered by the same two lecturers, and in both groups, the lecturers were visible via a video camera.

The participants had prior experience with the learning platform, whereas the webinar represented a novel and unfamiliar learning format within the Austrian Red Cross educational program.

The learning platform provided all participants, regardless of the study group, with access to in-depth materials, such as expert interviews with a psychiatrist, additional case studies, and supplementary information that could not be covered during training due to time constraints. This allowed even those learners seeking to deepen their understanding beyond the basic level to benefit from the program. Learners also received resources, including PowerPoint slides and audio material, which enabled them to review the content at their own pace after the training. In addition, the training incorporated both visual and auditory elements (e.g., PowerPoint slides and videos). The use of diverse communication methods, particularly within the webinar group, was actively encouraged, ensuring that learners with varying learning styles could effectively engage with the material.

### Questionnaires

The online questionnaire completed after the course included 13 questions on demographics, 11 questions examining the preference for and acceptance of online learning formats, 20 questions assessing intrinsic motivation, and 20 multiple-choice questions assessing the participants’ posttraining knowledge status (Fig. 1).

#### Acceptance of online learning, learning preferences and evaluation of further training

The participants evaluated further training on a scale from 1 to 5: 1 = very good, 2 = good, 3 = satisfactory, 4 = sufficient and 5 = deficient. The questions regarding paramedics’ acceptance of online teaching and learning preferences were scored via a 5-point Likert scale [1 (not at all true) to 5 (totally true)]. The 11 self-developed questions designed to assess acceptance of online teaching, learning preferences and evaluation of further training are included in the supplementary data (Additional file 1). A variable, designated “acceptance” (of the applied online teaching format), was calculated, which provided a summary score of Questions 3 to 6 in the supplementary data. The calculated variable “acceptance” included the four items that best represented the construct and exhibited the highest degree of internal consistency, as indicated by a *Cronbach’s alpha* value of.85.

#### Motivation

Intrinsic motivation, as well as perceived competence, perceived pressure, and perceived choice, were assessed by the respective subscales of the Intrinsic Motivation Inventory (IMI), which was developed by Ryan and Deci [23]. Each of the 28 items is evaluated on a 5-point Likert scale [1 (not at all true) to 5 (totally true)]. The IMI consists of several subscales, including interest/enjoyment (*Cronbach’s alpha =.*90*)*, perceived competence (*Cronbach’s alpha =.*79*)*, perceived pressure/tension (*Cronbach’s alpha =.*70*)*, and perceived choice (*Cronbach’s alpha =.*83*)*. Each subscale was analyzed separately. Sample items from each subscale include the following:

• Interest/Enjoyment: *“I really enjoyed the training”* (6 items).

• Perceived Competence: *“I believe that I am quite good at the tasks in the training.”* (5 items).

• Perceived Pressure/Tension: *“I felt pressured during the training.”* (5 items).

• Perceived Choice: *“I had the feeling that it was my choice to participate in the training.”* (4 items).

#### Knowledge test

The 20 multiple-choice questions included content that had previously been covered in the training course. The majority of the questions tested the ability to apply knowledge, whereas only a small number tested exclusively factual knowledge. The multiple-choice questions were optimized through a pretest with a smaller cohort. The discrimination indices demonstrated that the questions exhibited a high degree of discriminatory power. The mean discriminatory power was 0.45 across all the questions. Only one of the 20 questions exhibited an “acceptable” level of discriminatory power, with a discrimination index of 0.25. The optimal range for task difficulty, as proposed by Möltner et al., is between 0.4 and 0.8 [24]. The mean difficulty of the multiple-choice questions in this study was 0.63, indicating that the difficulty level was well suited. The total score was calculated from the 20 multiple-choice questions. The 20 multiple-choice questions, which were developed by the author, can be found in the appendix (Additional file 2).

### Data analysis

The data were analyzed through t tests, linear regressions, chi-square tests, ANCOVAs and mediation analyses to examine group differences, relationships between variables, and potential mediating effects. T tests were used to compare means between groups, regression analyses were used to assess the predictive relationships between variables, chi-square tests were used to examine categorical differences, ANCOVAs were used to control for covariates when comparing groups, and mediation analyses were used to explore indirect effects within the model. All analyses were conducted via SPSS (SPSS^®^ 29, IBM^®^, Armonk, USA). The assumptions of linearity, normality, and homoscedasticity were assessed, ensuring the appropriateness of the models. Mediation analyses were performed via the PROCESS macro by Hayes (Version 4.2, 2022), which uses ordinary least squares regression, yielding unstandardized path coefficients for total, direct, and indirect effects. Bootstrapping with 5000 samples together with heteroscedasticity-consistent standard errors was employed to compute confidence intervals and inferential statistics [25]. The effects were deemed significant on the basis of the *p* value threshold (*p* <.05). Multiple linear regression analyses were performed via a stepwise procedure that systematically included only those predictors that contributed the most to the model’s explained variance (*R*²). In this approach, variables were entered sequentially, and only those that significantly improved the model’s fit were retained, ensuring that the final model explained the highest possible amount of variance. Furthermore, key demographic variables such as age and educational level were included as covariates to control for their influence on the dependent variable. In all regression analyses, standardized beta values (*β*) are reported. To control for the accumulation of the alpha error rate resulting from multiple testing, the Bonferroni‒Holm procedure was applied to the 13 conducted tests. In this approach, *p* values were sequentially adjusted according to the Bonferroni‒Holm method and subsequently compared to the nominal alpha level of 0.05. Specifically, the smallest *p* value was multiplied by 13, the second smallest by 12, and so forth, with statistical significance determined for those tests yielding an adjusted p value (*p_adj*) below 0.05. The discrimination index (D) and severity of the multiple-choice questions were calculated in accordance with the methodology proposed by Möltner et al. [24].

## Results

### Participant characteristics

Out of the 5910 paramedics who were invited to take part in the course, 1044 participated in the study between January and February 2024 and completed the questionnaire. A total of 545 paramedics were included in the asynchronous teaching group (learning platform), whereas 499 paramedics were included in the synchronous teaching group (webinar) (Table 1). The participants in the study included both full-time paramedics and volunteers and those who work full-time and voluntarily in the EMS. More full-time paramedics and fewer volunteers completed the training via the online platform than through the webinar (*p* =.026, *Cramér’s V* = 0.08). In the webinar group (*N* = 499), 406 participants (81.4%) were volunteers, 62 (12.4%) were full-time paramedics, and 31 (6.2%) were both. In the learning platform group (*N* = 545), 406 participants (74.5%) were volunteers, 89 (16.3%) were full-time paramedics, and 50 (9.2%) were both. Additionally, the synchronous teaching group had a significantly higher level of education than the asynchronous group did (*p* =.035). The effect size was small (*Cohen’s d* = − 0.131, 95% CI [-0.253, − 0.010]), indicating a minor practical difference. On average, participants in the learning platform group had 12.62 years of education, whereas those in the webinar group had 12.92 years, with a 95% confidence interval for the mean difference of [-0.585, − 0.021]. Other demographic variables, such as age and prior experience in emergency medical services, did not differ significantly between the groups.

**Table 1:** Characteristics of the study participants

	asynchronous format	synchronous format	total
Gender			
Female	182	176	358
Male	362	323	685
Other	1	0	1
Age [in years]			
< 20	29	29	58
20–24	155	137	292
25–29	100	97	197
30–34	70	72	142
35–39	47	42	89
40–44	39	41	80
45–49	39	34	73
50–54	28	16	44
55–59	17	15	32
60–64	14	9	23
> 65	7	7	14
Country of origin			
Austria	535	486	1021
Germany	3	11	14
EU country	3	2	5
Country outside the EU	3	0	3
Duration of education [in years]	12.62 (± 2.20)	12.92 (± 2.43)*	12.76 (± 2.31)
Red cross employment status			
Voluntary	406	406	812
Full-time	89	62	151
Both	50	31*	81
EMS medical education			
EMT	430	399	829
Paramedics	115	100	215
Other medical education except EMS education [in years]	0.89 (± 1.33)	0.83 (± 1.32)	0.86 (± 1.32)
Experience in EMSs [in years]			
< 1	11	17	28
1–3	118	107	225
3–5	77	64	141
5–10	119	115	234
10–20	110	115	225
20–30	72	54	126
30–40	28	22	50
> 40	10	5	15
Number of hours working in EMSs per week [in hours]			
< 10	228	195	423
10–20	163	190	353
20–30	40	28	68
30–40	44	25	69
> 40	70	61	131

Values are given as the means (± SDs), medians (ranges) or absolute numbers

Significant group differences are marked with * *p* <.05

### Acceptance and preferences

**Fig. 2:**
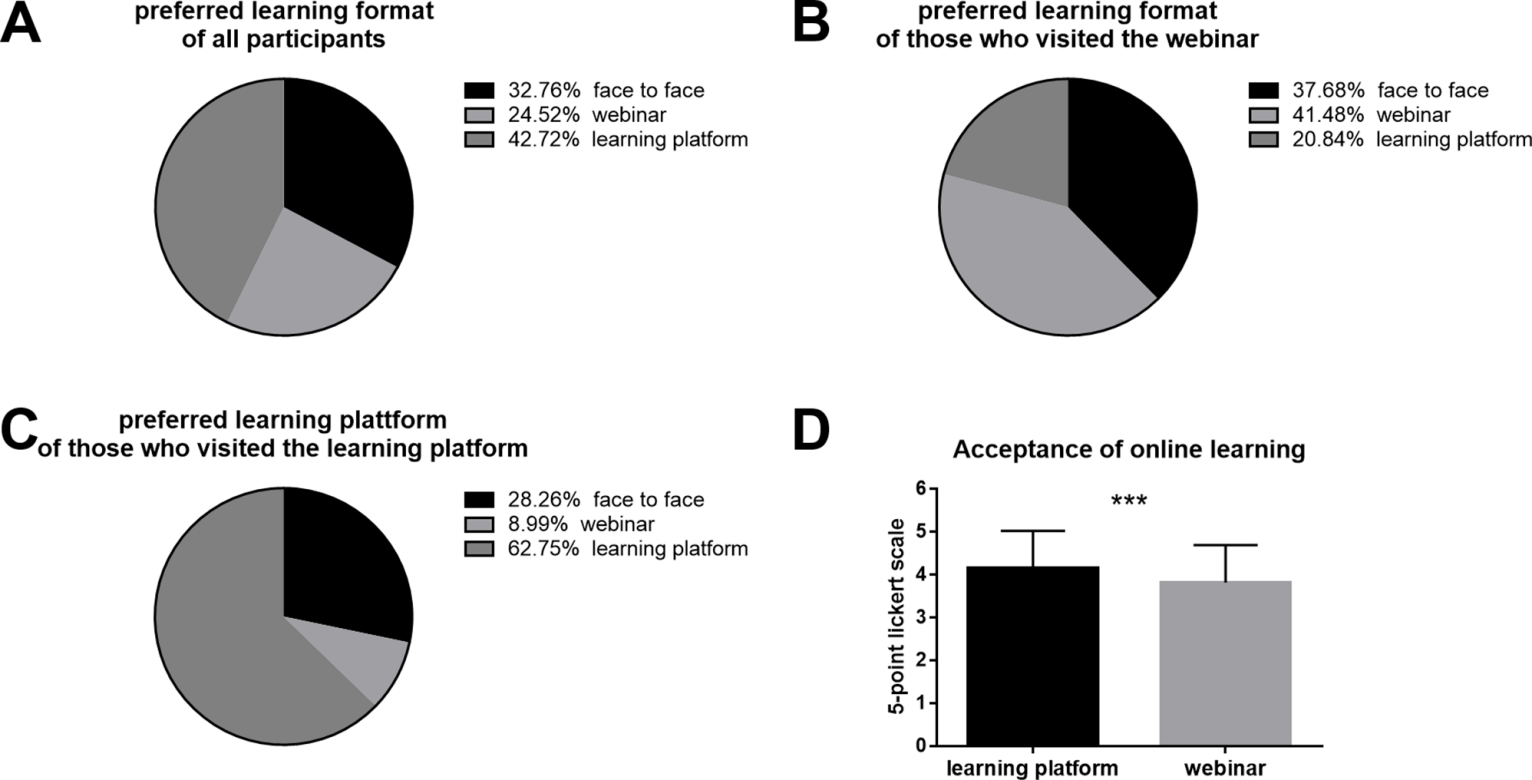
Acceptance of online learning: **A**: Preferred learning format of all participants in both study groups after they took part in the online learning session. **B**: Preferred learning formats of the participants who attended the webinar. **C**: Preferred learning format of the participants who completed the course on the learning platform. **D**: Acceptance of the online learning format, measured on a 5-point Likert scale. Comparison between the webinar and the learning platform

After either participating in the webinar training or training on the learning platform, the participants were asked about their preferred learning format. Two-thirds (67.2%) of the paramedics in Styria preferred online teaching over conventional face-to-face teaching (32.8% preferred this learning format) for further training. The learning platform was the most popular option overall, with 42.7% of the respondents preferring this format. In contrast, synchronous online teaching was the least popular learning format, with 24.5% of respondents choosing it (Fig. 2A).

A comparison of the two study groups indicated that the paramedics who participated in the webinar demonstrated a preference for the synchronous learning format, whereas those using the learning platform favored the asynchronous learning format (Fig. 2B and C). A chi-square test was used to compare learning format preferences while considering the most recently experienced learning format. The results demonstrated a statistically significant correlation between the training format and the preferred learning format (*χ²* [2] = 226.313, *p* <.001, *p_adj* = 0.013, *Cramér’s V* = 0.466).

Group comparisons revealed that the level of acceptance of the learning platform was significantly greater than that of the webinar (*M*_*LP*_ = 4.16 ± 0.87; *M*_*W*_ = 3.82 ± 0.88; *t* (1032) = 6.27; *p* <.001, *p_adj* = 0.013; *Cohen’s d* = 0.388, 95% CI [0.266, 0.511]) (Fig. 2D). The evaluation of the training in terms of Austrian school grades (scores from 1 to 5) was identical among participants using the webinar and the learning platform (*M*_*LP*_ = 1.98 ± 0.87; *M*_*W*_ = 1.94 ± 0.82; *t* (1042) = 0.689; *p* =.245, *p_adj* = 0.98). Therefore, the greater preference for the learning platform was not a consequence of a better evaluation of the training on the learning platform. Although the participants expressed a preference for the learning format they had previously used, overall, they preferred the learning platform.

Multiple linear regression analysis across all participants demonstrated that the following factors predicted the acceptance of the online teaching format: learning format (*β* = − 0.238, *p* <.001), perceived competence (*β* = 0.053, *p* =.012), evaluation of the training in school grades (*β* = − 0.094, *p* <.001), feeling of well-being during training (*β* = 0.187, *p* <.001), previous experience with online learning (*β* = 0.389, *p* <.001) and intrinsic motivation (*β* = 0.274, *p* <.001), (*F* (15, 1028) = 153.626; *p* <.001, *p_adj* < 0.013). The *R*^*2*^ for the overall model was 0.692 (adjusted *R*^*2*^ = 0.687), which indicated a high goodness-of-fit according to conventional benchmarks for explained variance.

### Knowledge status after training

**Fig. 3:**
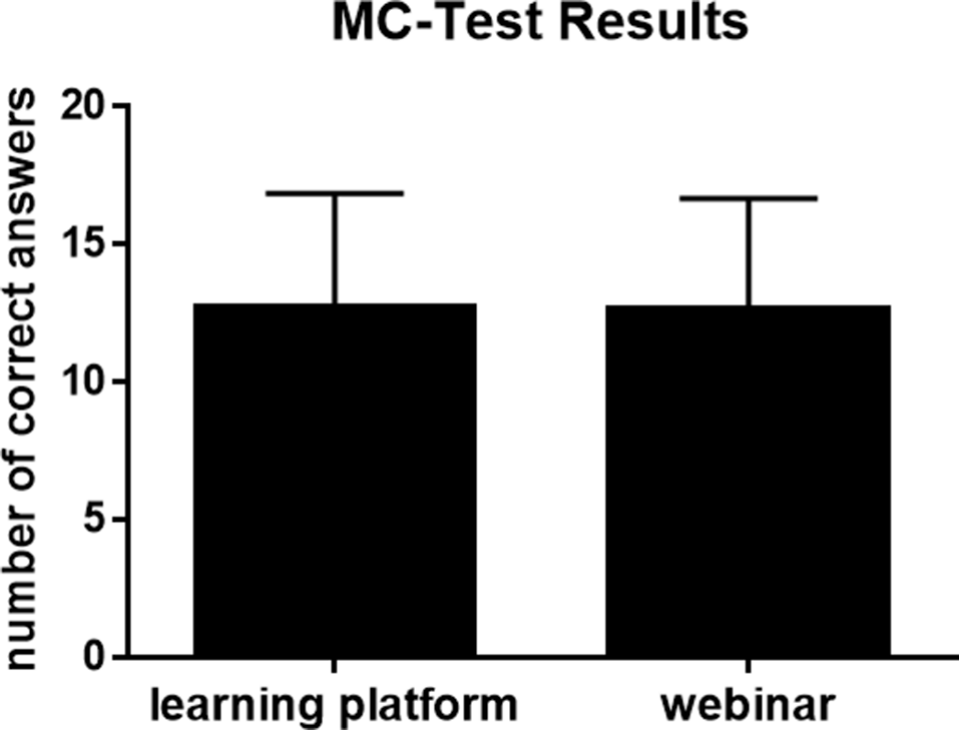
Results of the multiple-choice test, which was completed by all participants after further training. The participants were required to respond to 20 multiple-choice questions pertaining to the impact of the course. The figure illustrates the distribution of the results for each group, including the mean (12.6 points) and the standard deviation

When comparing the asynchronous and synchronous study groups, we found no significant differences in knowledge status after training according to the multiple-choice test (*M*_*LP*_ = 12.69 ± 4.17; *M*_*W*_ = 12.66 ± 4.01, *p* =.449, *p_adj* > 0.999) (Fig. 3).

Linear regression among all participants revealed that individuals with a higher level of education performed significantly better on the multiple-choice test (*β* = 0.406; *R*^*2*^ *=* 0.053; *F* (1, 1042) = 57.907; *p* <.001, *p_adj* < 0.013). After adjusting for the paramedics’ duration of education (highest educational qualification, in years) via ANCOVA, the multiple-choice results did not differ significantly (*F* (1,1041) = 0.398, *p* =.528, *p_adj* > 0.999, *η*^*2*^ = 0.000).

### Factors influencing the test results

Multiple linear regression analysis of the entire sample revealed the following factors (independent variables) as predictors of the test results (dependent variable): perceived competence (*β* = − 0.094, *p* =.005), pressure/tension (*β* = − 0.274, *p* <.001), duration of education (in years; *β* = 0.196, *p* <.001), experience in EMS (in years; *β* = − 0.109, *p* =.010) and medical education except from the EMS (in years; *β* = 0.090, *p* =.003), (*F* (13, 1030) = 17.262; *p* <.001, *p_adj* < 0.013). The *R*^*2*^ for the overall model was 0.179 (adjusted *R*^*2*^ = 0.169), which indicated a medium goodness-of-fit according to conventional benchmarks for explained variance.

### Intrinsic motivation

**Fig. 4:**
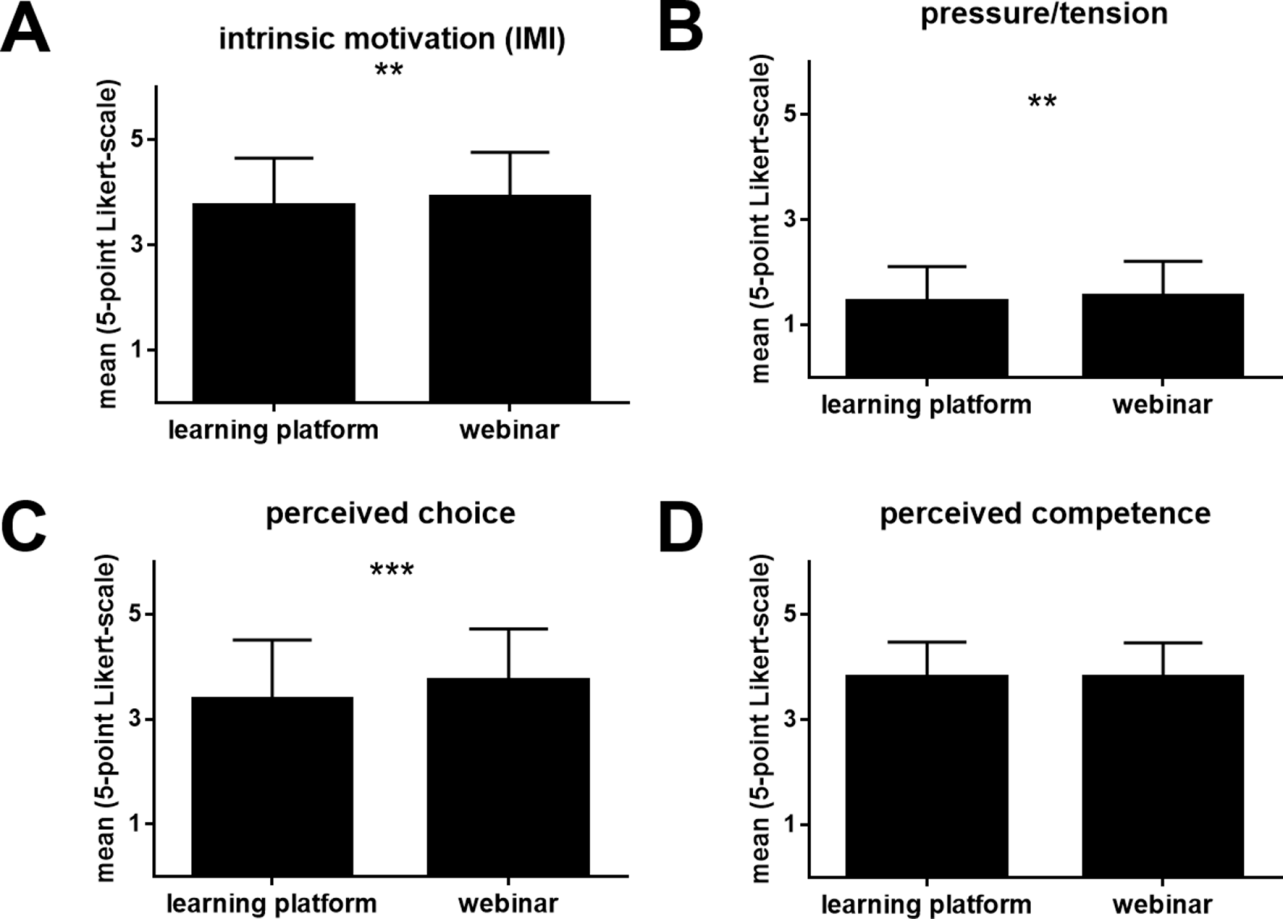
Intrinsic motivation interview (IMI). Comparison between the participants who completed training through the learning platform and those who took part in the webinar. Each item was scored on a 5-point Likert scale: **A**: Intrinsic motivation among the participants during further training. **B**: Perceived pressure and tension during further training. **C**: Perceived freedom of choice to participate in further training. **D**: Perceived competence of the participants with respect to the training content after they participated in further training

Compared with those who completed training through the learning platform, the paramedics who participated in the webinar presented a significantly greater level of intrinsic motivation (IMI; interest) (*M*_*LP*_ = 3.75 ± 0.90; *M*_*W*_ = 3.91 ± 0.84; *t* (1042) = -3.03; *p* =.001, *p_adj* = 0.013; *Cohen’s d* = − 0.188, 95% CI [-0.310, − 0.066]; Fig. 4A).

In addition, the webinar led to a significantly greater level of perceived freedom of choice (IMI perceived choice) than the learning platform did (*M*_*LP*_ = 3.29 ± 1.30; *M*_*W*_ = 3.73 ± 1.12; *t* (1042) = -5.71; *p* <.001, *p_adj* < 0.013; *Cohen’s d* = − 0.354, 95% CI [-0.476, − 0.231]; Fig. 4B).

However, the level of tension and pressure (IMI; pressure/tension) was significantly greater during webinar training than during training through the learning platform (*M*_*LP*_ = 1.44 ± 0.67; *M*_*W*_ = 1.56 ± 0.66; *t* (1042) = -2.75; *p* =.003, *p_adj* = 0.015; *Cohen’s d* = − 0.170, 95% CI [-0.292, − 0.048]; Fig. 4C).

The two learning formats were found to be equally effective in terms of perceived competence (*M*_*LP*_ = 3.80 ± 0.68; *M*_*W*_ = 3.81 ± 0.66; *t* (1042) = − 0.203, *p* =.420, *p_adj* > 0.999; Fig. 4D).

### Influencing factors of intrinsic motivation

By examining the full dataset, we found that the following factors were identified as significant predictors of intrinsic motivation during online learning via multiple linear regression: learning format (*β* = 0.050, *p* =.043), competence (*β* = 0.064, *p* =.001), pressure/tension (*β* = 0.038, *p* =.037), perceived freedom of choice (*β* = 0.181, *p* <.001), evaluation of the training in school grades (*β* = − 0.416, *p* <.001), professional discussions (*β* = 0.092, *p* =.014), acceptance of the learning format (*β* = 0.244, *p* <.001) and well-being during the training (*β* = 0.159, *p* <.001), (*F* (15, 1028) = 180.932; *p* <.001, *p_adj* = 0.013). The *R*^*2*^ for the overall model was 0.725 (adjusted *R*^*2*^ = 0.721), which indicated a high goodness-of-fit according to conventional benchmarks for explained variance.

### Mediation analyses of intrinsic motivation

**Fig. 5:**
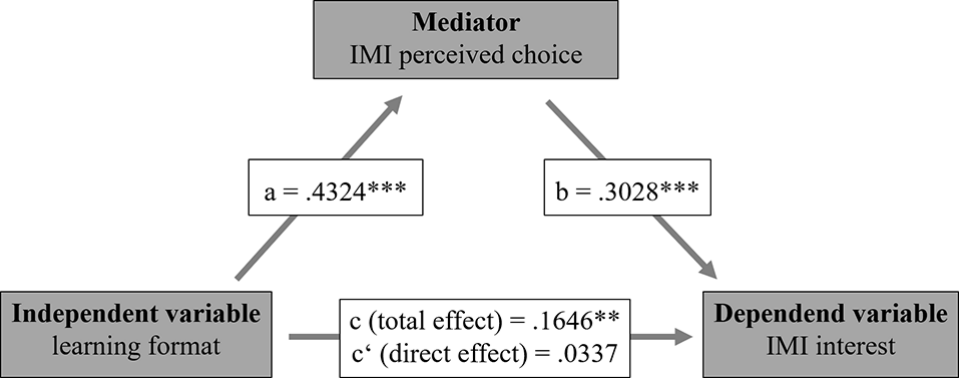
Mediation analysis of intrinsic motivation: The webinar group demonstrated greater intrinsic motivation than the learning platform group did. This relationship was fully mediated by perceived choice

A simple mediation analysis across the whole sample was performed to examine whether the learning format predicted intrinsic motivation and whether the direct path was mediated by perceived choice (Fig. 5). An effect of the learning format on intrinsic motivation was observed (*B* = 0.1646, *p* <.01). After adding perceived choice to the model as a mediator, the learning format significantly predicted perceived choice (*B* = 0.4324, *p* <.001), which in turn significantly predicted intrinsic motivation (*B* = 0.3028, *p* <.001). The relationship between learning format and intrinsic motivation was fully mediated by perceived choice, with an indirect effect (*ab)* of 1309 (95% *CI* [0.0849, 0.1809]). This finding indicates that the paramedics who took part in the webinar demonstrated greater intrinsic motivation than did those who completed training through the learning platform because of their greater perceived freedom of choice. Another mediation analysis was conducted to examine the relationship between perceived pressure/tension and intrinsic motivation. The findings of this analysis indicated partial mediation, with an indirect effect of -0.0102 (95% CI [-0.0253, -0.0009]). Mediation analysis was not performed for perceived competence, as no significant difference was found between the two learning formats for this variable.

## Discussion

The present study revealed three main findings: Although acceptance and intrinsic motivation were well above average in both conditions, acceptance of the learning platform was moderately greater and intrinsic motivation was slightly greater in the webinar group. No differences were found between the groups in terms of knowledge levels after training.

### Acceptance and preferences

The acceptance of the online learning format is a crucial determinant of its success, as a positive view enhances motivation, engagement and learning outcomes [13, 26]. Multiple linear regression analysis demonstrated that the learning format, intrinsic motivation, perceived well-being during training and previous experience with online learning are significant predictors influencing acceptance of the learning format.

### Technology acceptance model

The results revealed that the asynchronous learning format was more accepted than the synchronous learning format was, which might be explained by several factors. The Technology Acceptance Model (TAM) suggests that perceived usefulness and ease of use are two key determinants of acceptance of online learning formats [26]. The flexibility offered by asynchronous learning allows participants to control their own pace and schedule, which is one of the main advantages of this type of learning, increasing perceived usefulness [7, 26, 27]. In addition, asynchronous learning formats are easier to use because, to participate in webinars, participants have to meet higher technical requirements (e.g., a stable internet connection without interruptions, a quiet environment, microphone and camera). Additionally, the participants were already familiar with the learning platform, whereas the webinar was a new and unfamiliar learning format within the Austrian Red Cross educational program. This may result in the learning platform being perceived as more user friendly and easier to use by the participants. To increase the acceptance of webinars, providing technical support and training and minimizing the (technical) requirements to increase the ease of use are recommended.

### Previous experiences with online learning

Another finding of this study is that participants with positive experiences with online learning in the past had positive attitudes toward this mode of education, whereas those with negative past experiences demonstrated lower levels of acceptance. This finding is consistent with several psychological and educational theories, such as the TAM, experiential learning theory and social learning theory [26, 28, 29]. Positive experiences strengthen a cycle of openness and engagement with (online) learning, whereas negative experiences lead to avoidance and lower acceptance [28, 29]. The coronavirus disease 2019 (COVID-19) pandemic has significantly increased exposure to online learning, making these experiences even more impactful. Ensuring positive initial interactions with online learning is therefore crucial to prevent long-term negative attitudes. Otherwise, negative perceptions may become internalized by participants, thereby making it challenging to achieve a positive shift in their perspectives.

### Preferences for attended learning formats

The results also indicated that the learning platform was the preferred learning format, highlighting its flexibility and support for independent learning [7, 27]. Nevertheless, a more detailed examination of the preferences within the individual study groups revealed an important pattern: Participants demonstrated a clear tendency to favor the learning format they had just attended. Those who had participated in a webinar indicated a preference for synchronous learning formats, whereas those who had utilized the learning platform expressed a preference for asynchronous learning formats and indicated no desire to participate in webinars. This pattern can be explained by the mere exposure effect, a psychological concept that states that familiarity induces comfort and positive attitudes, which likely influence participants’ preferences [30]. It is therefore recommended that online teaching be used regularly, as this possibly reduces uncertainty and skepticism toward these teaching formats and potentially subsequently alters the preferences of participants.

### Knowledge status after training

In (online) education, learning success is an important measure of effectiveness. In this study, the efficacy of the learning process was assessed via multiple-choice questions, which has been demonstrated to be a proven method [31]. The calculated discriminatory power (mean = 0.45) and difficulty (mean = 0.63) of the multiple-choice questions were within the optimal range [24]. The finding that the test results were identical between the asynchronous and synchronous study groups is consistent with previous research, which suggests that both modalities are almost equally effective [32, 33]. Both learning formats have advantages and disadvantages. Asynchronous learning affords participants the flexibility to engage with the content at their own pace, which can reduce cognitive load, improve memorization and facilitate deep learning through repeated review [7, 34, 35]. However, synchronous learning enables real-time interaction, thereby enhancing learner engagement. The availability of immediate feedback and social interactions are essential for the achievement of optimal learning outcomes [7, 36]. The available evidence indicates that there is no notable, relevant difference in learning outcomes between asynchronous and synchronous learning methods [32, 33]. The success of these learning methods is more dependent on the quality of the content and the engagement strategies employed than on the educational mode itself [37]. Given that both teaching formats are equally efficient, educators and educational institutions can select the format that is most appropriate according to the participants’ requirements and preferences. Furthermore, financial considerations may also be a relevant factor in this decision.

### Influences on test results

Multiple linear regression analysis revealed that participants’ perceived competence and pressure, level of education and experience in EMSs are significant predictors of their knowledge status after training. Linear regression revealed that participants with higher (medical or nonmedical) education levels achieved better test results, which is consistent with previous research: individuals with advanced education tend to employ more effective learning strategies, exhibit stronger self-regulation, and engage more readily with complex material, which all enhance their comprehension and performance [38, 39]. These findings suggest that learners with higher educational backgrounds have a distinct advantage in online environments. Therefore, additional support should be provided to learners with lower education levels to address their specific challenges.

### Pressure and tension

The study revealed a negative correlation between heightened pressure and test results, which is consistent with previous research showing that stress impairs cognitive performance and retention [15]. Cognitive load theory suggests that stress increases the cognitive load, limiting the resources available for processing new information [34, 35]. Consequently, learners may experience difficulties in understanding and recalling the material, which may result in reduced learning performance. Furthermore, elevated levels of pressure can result in a reduction in intrinsic motivation, in turn leading to lower levels of ‘deep learning’ [17, 40], which is supposed to foster critical thinking, deep understanding, and long-term cognitive development [40, 41]. Asynchronous learning, which offers more flexibility and control, typically lowers stress levels, whereas synchronous settings may intensify pressure and tension, particularly for learners who feel unprepared or anxious in in-person situations or when communicating with strangers. This added pressure can have a negative effect on performance [15]. To improve learning outcomes in both types of learning environments, online courses should focus on reducing unnecessary stress and creating a positive, low-pressure learning atmosphere. This strategy may assist learners in optimizing their cognitive resources and thereby achieving enhanced learning outcomes.

### Experience in EMSs and perceived competence

The study revealed that increased experience in EMSs is associated with poorer knowledge levels after training, contrary to the expectation that more experience improves performance. This may be explained by experienced individuals having a fixed mindset, resisting new information and adhering to established routines that hinder learning [42]. Furthermore, cognitive biases might be involved, where prior knowledge interferes with the adoption of new strategies, leading to suboptimal performance [43]. Additionally, individuals with high perceived competence also performed worse on the multiple-choice test, likely because of overconfidence. Although self-efficacy is typically associated with superior learning performance, inflated perceptions of competence result in overconfidence. This phenomenon, known as the Dunning–Kruger effect, leads such individuals to underestimate the need for further learning and adaptation [44]. These findings highlight the importance of promoting continuous learning and encouraging adaptability, regardless of experience or perceived competence. Further training should also address the gap between self-perception and actual competence to improve engagement.

### Intrinsic motivation

Intrinsic motivation has been identified as a key factor in successful learning, particularly in online environments where learners must self-regulate their engagement [17, 18]. Multiple linear regression analysis demonstrated that the evaluation of the training (in terms of school grades), perceived autonomy, acceptance of the learning format and perceived well-being are significant predictors of intrinsic motivation.

This study revealed that participants in the webinar group demonstrated higher levels of intrinsic motivation than those in the learning platform group did. This finding is supported by previous research, indicating that interactive learning environments significantly enhance perceived learning and satisfaction [45]. The higher level of motivation can be attributed to the interactive nature of webinars, which enables real-time engagement and fosters a sense of relatedness among learners. Social connectedness helps learners feel supported and valued by their peers, which is crucial for an effective learning process [17]. This illustrates the importance of establishing an engaging learning environment. While webinars naturally support intrinsic motivation, asynchronous learning platforms should also incorporate interactive features, such as discussion forums, Q&A sessions and gamification elements.

### Self-determination theory

In accordance with SDT, the three most significant factors that contribute to the development of intrinsic motivation are autonomy, competence and relatedness [17–19].

### Perceived choice

The study revealed that participants in the webinar group perceived greater autonomy than those in the learning platform group did. This was an unexpected result, as asynchronous learning formats are typically perceived as more flexible in terms of time management. Nevertheless, several factors may explain this result. Autonomy, defined as control over one’s decisions and actions, is a key concept in SDT. When learners perceive greater choices in terms of how they interact with learning material, their motivation tends to increase [17]. Webinars offer more real-time interaction, allowing learners to engage more actively, ask questions, contribute to discussions, and assume a more active role in their learning experience [45]. This interactivity not only promotes a heightened sense of involvement and autonomy, as learners feel more empowered to control the direction of their learning process but also signals to learners that their individual learning process is valued by the instructor, who takes the time to address their questions and provide feedback. The feeling that their learning process matters can further enhance motivation and engagement. In contrast, asynchronous platforms often follow a predetermined structure with less spontaneous interaction where learners must work through fixed content. These findings support the idea that autonomy involves not only time flexibility but also the ability to influence the learning process [46]. When learners feel that they have control over their learning process, they are more likely to engage in tasks out of personal interest rather than because of external pressure, which in turn leads to greater intrinsic motivation. The results of the study also reveal that intrinsic motivation is fully mediated by the sense of autonomy experienced by learners, explaining the higher intrinsic motivation in synchronous formats. This finding is consistent with those of the SDT [17]. It is recommended that educators design learning environments that offer learners more choices, even in asynchronous formats, to increase motivation and engagement. At the same time, it is important to maintain a balance between autonomy and guidance to avoid overwhelming learners with too many choices or a lack of direction.

### Perceived competence

The study revealed no statistically significant difference in perceived competence between participants in the synchronous learning group and those in the asynchronous learning group. The perceived competence of learners can be defined as their belief in their ability to successfully complete tasks or master material [17]. The identical competence levels between the groups indicate that both formats presented equally challenging content. This suggests that perceived competence may be more influenced by the complexity of content, the support provided and participants’ self-efficacy than by the learning method [47]. Learners who participate in training through both types of learning formats can feel equally capable if they receive adequate support and have strong self-efficacy, as both formats can foster competence through clear goals and progress evaluation.

### Perceived pressure and tension

As mentioned above, the study also demonstrated that the paramedics who participated in the webinar experienced more pressure and tension than those who completed the training on the learning platform did, likely due to the synchronous nature of webinars. Webinars often require active participation, such as answering questions or contributing to discussions in front of others, which can increase stress for some learners [48]. Real-time interactions, particularly when speaking via microphones or cameras or to unknown individuals, can induce performance anxiety and social pressure. Additionally, in contrast to asynchronous platforms, webinars demand continuous attention without the flexibility of repeated review or to take breaks, which can lead to a sense of being under pressure or overwhelmed, particularly during longer sessions. This highlights the need to reduce stressors in synchronous formats, e.g., by allowing breaks, providing enough time to answer questions, and offering options for participation, such as using the chat, turning off the camera, or opting out of speaking. Despite experiencing heightened levels of pressure and tension in comparison to participants on the learning platform, no significant differences were observed in the knowledge test.

### Relationship between intrinsic motivation and evaluation of the training

Furthermore, the study revealed a correlation between better course evaluations and elevated levels of intrinsic motivation among the participants. One potential explanation for this is that intrinsically motivated participants tend to engage more deeply with the learning material and perceive courses as well-structured and valuable, which in turn leads to better evaluations [49]. Conversely, the structure, quality and relevance of the course may play a central role in fostering intrinsic motivation [50–52]. A reciprocal relationship may exist where highly motivated learners engage more deeply and feel more satisfied, leading to better evaluations, while well-designed courses can increase motivation, creating a positive feedback loop. This emphasizes the importance of fostering intrinsic motivation through the use of a well-structured online course.

### Limitations

This study has several limitations that should be considered when the results are interpreted. Primarily, the randomization process and the characteristics of the sample must be recognized as limitations. The learning platform group consisted of more full-time paramedics and fewer volunteers than did the webinar group. Furthermore, participants in the synchronous teaching condition presented a significantly greater level of education, with an average of 12.92 years, than did those in the asynchronous group, with a 95% confidence interval for the mean difference of [-0.585, − 0.021]. While a higher education level could be expected to enhance test results, no such advantage was observed in the webinar group. This finding suggests that the asynchronous format may be effective for learning regardless of educational background. A further significant limitation is the utilization of a single posttest design for the evaluation of knowledge. The knowledge assessment was only conducted after the training, which raises questions about the extent to which the observed results reflect actual learning gains or preexisting knowledge. The use of a pre- and posttest design would provide more insight into individual progress and knowledge retention over time. Future research should address this issue by incorporating baseline assessments and follow-up testing to determine long-term learning effects. Furthermore, the response rate in this study was 17.6%, which gives rise to concerns about selection bias. It is possible that individuals who were particularly engaged or interested in the training were more likely to participate, potentially biasing the results. The extent to which the findings can be generalized to the broader target population remains uncertain, emphasizing the need for replication studies with higher response rates. In addition, the study was conducted within the Austrian Red Cross educational system, which may limit the generalizability of the findings to other emergency medical systems or different professional learning environments. The effectiveness and acceptance of different online learning materials may be influenced by variations in institutional structures, technological infrastructure, and instructional design. Furthermore, given that the participants were largely from Austria, cultural and educational differences may affect the applicability of the findings to other countries or contexts. Moreover, the study sample was predominantly male, constituting 65.6% of the total participants. This gender imbalance should be considered when interpreting the results, as learning preferences and motivational factors may differ between participants of different genders. Subsequent studies should aim to replicate these findings in different settings to increase their external validity. Despite the standardization of content, instructors and learning objectives were standardized for both study groups, and the inherent differences between synchronous and asynchronous formats cannot be fully eliminated. Synchronous and asynchronous learning environments are characterized by distinct instructional dynamics, including group interaction, learner autonomy, and temporal structure. These differences may have influenced participants’ perceptions and experiences and may limit the direct comparability of the two conditions. However, this limitation is characteristic of real-world study designs that aim to reflect authentic learning environments. It is recommended that future research build on these findings by using more controlled experimental settings or by isolating instructional style from the mode of delivery. Furthermore, the present study examined only one specific implementation of synchronous and asynchronous formats, specifically a live webinar including breakout rooms, rather than a learning platform with recorded lectures and case examples. Consequently, it is not possible to generalize the findings to all synchronous and asynchronous instructional designs, as a wide range of possible approaches (e.g., formats incorporating gamification, adaptive learning, or collaborative tools) may yield different outcomes.

## Conclusion

This study offers insights into the distinctions between asynchronous and synchronous online teaching formats, with a focus on acceptance, intrinsic motivation, and knowledge levels after training.

The findings of this study indicate that recorded lectures on a learning platform show a significantly higher level of acceptance due to its flexibility and ease of use, which is in agreement with previous research. The participants in the webinar group expressed a preference for synchronous learning formats as their favorite learning format, whereas those who attended the learning platform expressed a preference for the asynchronous learning format. Furthermore, learners’ prior experiences with online learning have a considerable effect on their acceptance of the learning format, emphasizing the significance of positive initial exposure. However, the synchronous format, despite being less accepted, has been shown to foster higher levels of intrinsic motivation, which is likely driven by its interactive nature and the sense of autonomy it affords. Furthermore, the results indicate that intrinsic motivation is fully mediated by perceived choice, suggesting that enhancing learner autonomy could enhance engagement across both formats. The results demonstrated no significant difference in test results between the two formats, indicating that both methods are equally effective. This is consistent with the findings of previous studies, which indicate that the quality of the content and the engagement strategies employed are more important for learning outcomes than the format in which the content is delivered.

Future research could address the unequal distribution of participant types across groups and increase the response rate to minimize selection bias. Furthermore, pre- and posttesting with follow-up assessments should be employed to measure learning gains accurately, and controlled designs should be used to isolate instructional dynamics (e.g., interactivity and pacing) from the delivery mode, thereby enhancing generalizability and internal validity. In future research, a more diverse spectrum of synchronous and asynchronous learning formats should be assessed (e.g., online learning incorporating gamification elements). Moreover, it would be advantageous to investigate other aspects, such as the influence of the camera in webinars and the perceived relatedness between the participants.

In conclusion, while both teaching formats offer distinct advantages and disadvantages, educational institutions should carefully consider the specific needs and preferences of learners when selecting a format. Online teaching methods must be continuously adapted and improved to ensure the optimal effectiveness of future educational programs.

## Electronic supplementary material

Below is the link to the electronic supplementary material.

Supplementary Material 1

Supplementary Material 2

## Data Availability

The data analysed in this study can be made available by the corresponding author upon reasonable request.

## Abbreviations

LP: Learning platform, asynchronous learning format
W: Webinar, synchronous learning format
EMS: Emergency medical service
EMTs: Emergency medical technicians
IMI: Intrinsic motivation inventory
SD: Standard deviation
*M*_*LP*_: Mean (learning platform)
*M*_*W*_: Mean (webinar)
TAM: Technology acceptance model
