# Supplementary material for: Randomized controlled trial of asynchronous vs. synchronous online teaching formats: equal knowledge after training, greater acceptance and lower intrinsic motivation through asynchronous online learning
Source: BMC Med Educ. 2025 Jun 19;25:850. doi: 10.1186/s12909-025-07481-4 (PMC12180198; doi:10.1186/s12909-025-07481-4)
Supplement: Supplementary file 1 — Supplementary Material 1 [file 12909_2025_7481_MOESM1_ESM.docx]

| **Question** | **Answer** |
| --- | --- |
| 1. I rate the further training as follows: | 1 = Very good  2 = Good  3 = Satisfactory  4 = Sufficient  5 = Not sufficient |
| 1. If I had to choose a learning format, I would choose the following: | [1] Classroom teaching on site  [2] Webinar via Microsoft Teams (synchronous online learning)  [3] Online platform of the Austrian Red Cross (asynchronous online learning) |
| 1. "I consider the online learning format utilized to be a suitable alternative to traditional face-to-face teaching for further training." 2. "The utilized online learning format enabled me to engage intensively with the content." 3. "The utilized teaching format helped me to learn independently." 4. With the online learning format, I was able to organize my time more flexibly (than with other learning formats).” 5. “I have had good experiences with online teaching in the past.” 6. “I had enough opportunities to ask questions.” 7. “There were enough opportunities for exchanges with colleagues.” 8. “There were enough opportunities for professional discussions.” 9. “I felt comfortable during the training.” (subsequentially referred to as “well-being”) | *Likert-Scale:*  [1] not at all true [2] rather not true [3] partly, partly [4] rather true [5] totally true |
