# Supplementary material for: Randomized controlled trial of asynchronous vs. synchronous online teaching formats: equal knowledge after training, greater acceptance and lower intrinsic motivation through asynchronous online learning
Source: BMC Med Educ. 2025 Jun 19;25:850. doi: 10.1186/s12909-025-07481-4 (PMC12180198; doi:10.1186/s12909-025-07481-4)
Supplement: Supplementary file 2 — Supplementary Material 2 [file 12909_2025_7481_MOESM2_ESM.docx]

**20 Multiple-choice questions**

(correct answer = **bold** and *italics*)

1. **Which statement best fits a patient with an affective disorder?**
2. “I think aliens are following me.”
3. ***"Other people always disappoint me, and there’s no point in trying because I can’t change their stupidity."***
4. "I am very afraid that people think I’m crazy or that they find me embarrassing. That’s why I hardly go out anymore."
5. "I lost my job because I can’t get out of bed in the mornings after drinking too much again."
6. "I suddenly couldn’t breathe, and my heart started racing like crazy, even though there was no reason."
7. **What is true about anxiety disorders?**
8. Panic disorders are usually chronic.
9. Specific triggers (e.g., spiders) are the cause of anxiety in people with panic disorders.
10. Panic disorders are the least common form of anxiety disorder encountered in emergency services.
11. Anxiety disorders rarely lead to impairments in everyday life.
12. ***People affected by anxiety disorders often fear going crazy, dying, or losing control.***
13. **You are called to attend to a 5-year-old girl in the EMS.** **The girl has repeatedly told her mother that her grandmother has hurt her in her private parts. She is withdrawn and shy and refuses to be examined by you. The mother is concerned but does not want to transport the girl to the hospital, as she doesn’t want to upset her mother-in-law with the allegations.
    Which statement is correct?**
14. I should take the child to a hospital against the mother’s wishes owing to my legal duty to report such abuse.
15. I should try to confront the grandmother to substantiate the suspected child abuse.
16. There is a perpetrator profile and a catalog of symptoms that allow doctors to reliably recognize abuse cases.
17. ***Even if there is only suspected child abuse, I must inform the police.***
18. Changes in the child (e.g., aggressive behavior, sadness, withdrawal) are usually not warning signs but normal mood fluctuations in children and adolescents.
19. **The right to decline medical treatment or transport exists when… (multiple choice)***Indicate if each statement is true or false.*

***1. ... the adult's representative (responsible for medical matters) agrees.***

***2. ... the patient is capable of insight and judgment.***

***3. ... the legal representative agrees for children under 14 years old.***

***4. ... the form for declining treatment is signed voluntarily***

1. **Which of these statements is stigmatizing toward mentally ill people?**
2. “Child poverty is a risk factor for psychiatric disorders.”
3. “When dealing with patients with acute psychosis, it’s best to keep some distance and have a way to retreat.”
4. ***“Mental illnesses are incurable.”***
5. “Patients with acute psychosis are not fully capable of making judgments.”
6. “Patients with schizophrenia experience recurring psychosis.”
7. **Hostility and euphoria are most likely found in people with:**
8. Depression
9. Panic Disorder
10. ***Mania***
11. Personality Disorders
12. Addiction
13. **Which statement is true of schizophrenia?**
14. People with schizophrenia have split personalities.
15. Schizophrenia is incurable due to its severity.
16. ***Schizophrenia is a severe disorder of thought and perception.***
17. Patients with schizophrenia exhibit significant aggression and anger and are therefore considered dangerous.
18. Individuals with schizophrenia are statistically very often criminals, so paramedics should be especially concerned about their safety.
19. **Which of the following symptoms is the main symptom of depression?**
20. ***Lack of interest***
21. Reduced self-esteem
22. Suicidal thoughts
23. Sleep disturbances
24. Feelings of guilt
25. **You come to examine a 29-year-old patient with paranoid schizophrenia in the EMS.**

**The patient is hearing voices and is suffering from poisoning delusion.**

**What is the best way to handle the patient in the acute phase?**

1. I try to build trust and convince him that he is not being poisoned.

2. I support the patient by telling him I wouldn't drink the water either.

***3. I accept the fear of being poisoned as the patient's subjective reality.***

4. I drink a beverage in front of the patient so he can see that I am not poisoned.

5. I avoid talking to the patient to avoid further confusion.

1. **In the rescue service, you see a 24-year-old patient who is convinced that he has been abducted by aliens. As he knows that his abductors will return in a different form, he is very suspicious and is very reluctant to let you into his flat.
   You suspect the patient has paranoid schizophrenia and offer to admit him to a psychiatric clinic immediately. However, the patient refuses treatment.**

**Under what circumstances may the patient be taken directly to the hospital, even against his or her will?**

1. The patient is a hunter and has firearms in the house.
2. A legal guardian has been appointed for the patient.
3. The patient’s parents provide consent for the treatment.
4. ***The patient threatens to kill intruders in his house.***
5. The patient drinks only self-filtered water because of fear of poisoning.
6. *(Continuation)*

**What symptoms can be observed in the 24-year-old patient?**

1. Hallucinations
2. ***Delusions***
3. Depersonalization
4. Apathy
5. Depression
6. *(Continuation)*

**How can you attempt to de-escalate the situation?**

1. I trigger the “silent emergency alert.”
2. I avoid eye contact and refrain from making clear demands or requests to the patient.
3. ***I maintain a distance of about two arms’ lengths from the patient and avoid physical contact.***
4. I show the patient that I, as a paramedic, am in control.
5. I tell the patient that there are no aliens and that he is experiencing acute psychosis.
6. **A 45-year-old patient reports,** **“For the past six weeks, I have been incredibly tired. Everything takes twice as much effort as before. Often, I must stop working to rest because of exhaustion. Nothing brings me joy anymore. Even yesterday, when my family celebrated my birthday, I felt nothing inside. I can’t even cry anymore. It’s as if I’m empty inside. In addition, then there’s this constant feeling that I might be guilty of something, though I can’t pinpoint why. I know I haven’t done anything bad, yet the feeling persists.”**

**What is your probable diagnosis?**

1. Schizophrenia
2. ***Affective disorder***
3. Panic disorder
4. Obsessive‒compulsive disorder
5. Schizoid personality disorder
6. *(Continuation)*

**What symptoms can be observed in the 45-year-old patient?**

1. Affective lability
2. Loss of self-confidence
3. Concentration issues
4. Sleep disturbances
5. ***Lack of motivation (listlessness)***
6. **A 75-year-old patient with early-stage Alzheimer’s disease tells you during transport that life feels increasingly burdensome and that she sometimes wishes she wouldn’t wake up in the morning. When you ask if she has ever thought about taking her own life, she denies it. How do you assess the patient’s suicidality?**
7. No suicidality
8. Acute suicidality
9. Parasuicidal behavior
10. ***Passive suicidality***
11. Active suicidality
12. *(Continuation)*

**What is true regarding suicidality?**

1. Suicide is rarely preceded by warning signs.
2. When affected individuals become calmer, one can assume that suicidality is decreasing and that the person’s condition is improving.
3. The decision to commit suicide is usually a free choice.
4. ***The patient in the case above shows serious suicidal tendencies.***
5. As a paramedic, it’s best not to address suicidality, as only trained psychiatrists, psychologists, and psychotherapists should do so.
6. **A 35-year-old patient calls emergency services due to sudden shortness of breath, abdominal pain, and chest tightness.**
   **When you arrive, she is trembling and sweating, but the other physical symptoms have subsided. Earlier in the day, she saw her doctor for the same symptoms, but no physical cause was found.**

**Which two preliminary diagnoses are most likely given this symptom pattern?**

***Please select 2 answers!***

1. Severe depressive episode with somatic syndrome
2. ***Panic disorder***
3. Obsessive‒compulsive disorder
4. Phobic disorder
5. ***Acute coronary syndrome***
6. *(Continuation)*

**What is your next course of action?**

1. I transport the patient to a psychiatric department, as the symptoms seem to have no physical cause.
2. I leave the patient at home, as her condition has clearly improved upon arrival, and transport to the hospital no longer seems necessary.
3. ***I transport the patient to an internal medicine department.***
4. The patient is experiencing a psychosomatic condition and should schedule an appointment in a psychosomatic or psychiatric department.
5. I advise the patient to consult her doctor again to discuss the next steps.
6. **A 37-year-old patient calls emergency services because his girlfriend left him.** **He reports that she left because he drinks too much and had been encouraging him to cut back for some time. He recently lost his driver’s license for driving under the influence. During the conversation, it’s apparent that he is intoxicated, and you notice several empty bottles in his apartment. On weekends, he says he drinks approximately 7–8 bottles of beer in the evening, although he claims it wouldn’t be a problem to stop after just one. He doesn’t think alcohol is an issue, as he still manages to get to work on time every morning and loves fishing. He is now considering going through detox to win his girlfriend back.**

**Which sign of addiction is present in the 37-year-old patient?**

1. Difficulty regulating substance use
2. ***Continued use despite negative consequences***
3. Craving (= strong desire)
4. Prioritization of use over other activities
5. Withdrawal symptoms
6. *(Continuation)*

**What actions do you take, and what is your next course of action in the case described above?**

1. I immediately transport the patient to a psychiatric department for detoxification, as he has requested.
2. I transport the patient to a nonpsychiatric department due to withdrawal symptoms.
3. I immediately call the police for my own protection.
4. I transport the patient to an internal medicine unit, as psychiatric departments generally do not accept intoxicated patients.
5. ***The patient should schedule an appointment at a psychiatric clinic (Centre for Addiction Medicine).***
